# Effect of Different Cross-Fostering Strategies on Growth Performance, Stress Status and Immunoglobulin of Piglets

**DOI:** 10.3390/ani11020499

**Published:** 2021-02-14

**Authors:** Xiaojun Zhang, Meizhi Wang, Tengfei He, Shenfei Long, Yao Guo, Zhaohui Chen

**Affiliations:** State Key Laboratory of Animal Nutrition, College of Animal Science and Technology, China Agricultural University, Beijing 100193, China; zhangxiaojun14@163.com (X.Z.); meizhiwang@cau.edu.cn (M.W.); hetengfei@cau.edu.cn (T.H.); longshenfei@cau.edu.cn (S.L.); guoyaocau@163.com (Y.G.)

**Keywords:** cross-fostering, piglets, growth performance, stress status, immunoglobulin

## Abstract

**Simple Summary:**

Piglet survival in large litters can be increased if surplus piglets are cross-fostered to smaller litters, exploiting surplus teats in these sows. We aimed (1) to investigate the effect of cross-fostering piglets of different birth weights into new litters on the growth performance of piglets; (2) to determine the effect of cross-fostering piglets of different ages on the growth performance, stress and immunity of these piglets. Cross-fostering on day 2 after birth reduced average daily gain (ADG) in high birth weight (HBW) piglets. Late cross-fostering on day 7 after birth decreased ADG, affected the teat order and increased the cortisol level of piglets. Therefore, these results provide suitable cross-fostering strategies for improving cross-fostering piglets’ welfare.

**Abstract:**

The effect of different cross-fostering strategies on the growth performance, stress and immunity of piglets was investigated in this study. In the first experiment, a total of 20 litters (i.e., 20 sows) and 120 piglets were classified into one of six treatments in a 2 × 3 factorial arrangement. The treatments consisted of piglets without or with cross-fostering and different birth weights (low birth weight, LBW; intermediate birth weight, IBW; high birth weight, HBW). The weaning weight (WW) and average daily gain (ADG) of LBW piglets and IBW piglets were not significantly different between the not cross-fostered (NC-F) group and the cross-fostered (C-F) group. There was a higher (*p* < 0.05) ADG in the control piglets compared with the cross-fostered piglets. This effect on ADG was only seen in the HBW piglets. In the second experiment, six sows with a similar body condition and farrowed on the same day were selected. Three female piglets with a birth weight of 0.6–0.85 kg were selected from each litter as experimental piglets. Eighteen piglets were grouped into three treatments: (1) not cross-fostered (NC-F1), (2) cross-fostered at 36–48 h after birth (C-F1), (3) cross-fostered at day 7 after birth (C-F2). The growth performance of NC-F1 and C-F1 piglets was higher than C-F2 piglets (*p* < 0.05), and the suckling positions of NC-F1 and C-F1 piglets on days 8, 12, 16 and 20 were more forward than the C-F2 piglets (*p* < 0.05). Plasma cortisol (COR) concentrations of NC-F1 and C-F1 piglets were lower than C-F2 piglets (*p* < 0.05). A significant negative correlation was observed between BW at day 21 and plasma COR concentration. In conclusion, cross-fostering within 24 h of birth has adverse influences on the ADG of HBW piglets, while it has no negative effect on the ADG of LBW and IBW piglets. Moreover, for IBW piglets, late cross-fostering (i.e., on day 7 after farrowing) has negative impacts on the growth performance and teat order of piglets, and it increases the cortisol level of piglets.

## 1. Introduction

With the improvement of gene breeding technology and management practices, the number of surviving piglets per sow per year is increasing. When piglet numbers exceed available functional teats, piglets could have less milk intake, which could lead to a high risk of mortality [1,2]. Large litter size has negative animal welfare impacts on piglets and sows [2,3]. Therefore, some management strategies should be applied to reduce mortality and improve animal welfare for piglets in commercial farms. Cross-fostering is a management practice that transfers extra piglets from large litters to smaller litters, so that the sows with more functional teats can be put to good use [4,5,6]. Some authors have studied the effects of cross-fostering on the mortality and growth performance of piglets [7,8]. Other studies show that the age/stage of lactation and body weight/size of piglets affect the efficiency of cross-fostering [9,10].

Good maternal behavior and high milk production of sows have a positive effect on the growth performance of piglets [1,11]. Therefore, sows with good maternity and lactation capacity should be selected to improve the efficiency of cross-fostering. Small piglets with large litter mates spend more time competing for teats [12], thus, fostered piglet size is also an important factor for cross-fostering. Ferrari et al. [13] found that heavy birth weight fostered piglets had a higher weight at days 14 and 20 pre-weaning than light birth weight fostered piglets. Large piglets that were fostered in mixed litters consisting of equal numbers of light-weight and heavy-weight piglets had a greater growth rate than large piglets, but the growth performance of small fostered piglets in uniform litters was significantly increased [14]. However, Souza et al. [15] reported that small piglets had a similar body weight during lactation, regardless of being mixed with piglets of higher weights or not. Similar-sized piglets may face more competition [16], and homogeneous litters with only small piglets may not fully stimulate the breast, resulting in reduced milk production. Therefore, the body weight of small piglets that were grouped with large piglets in mixed litters could be improved because heavier piglets are able to stimulate the teats to remove more milk from the mammary glands [17]. There is still controversy about the effect of cross-fostering on the performance of piglets with different birth weights [1].

Teat order is established by piglets in early lactation [18], and cross-fostering should be completed as early as possible during this time [5]. Teat order was not established when piglets were fostered at 12–24 h after birth and they could absorb more colostrum immunoglobulins [5,19,20]. There was little impact on the weight gain of piglets when they were fostered at 12 h after parturition [12]. Heim et al. [5] showed that cross-fostering at 14–24 h after birth had no negative effect on the growth performance and survival rate of fostered piglets. It was shown that cross-fostering at 48 h after birth had no adverse effect on growth performance, but fostered piglets had a higher risk of death [21]. In practice, some piglets fall behind through lactation so that they may be subjected to late cross-fostering. Late cross-fostering means fostered piglets will face more competition because the teat order has been established. Some studies indicated that late cross-fostering reduced the body weight gain of piglets [22,23]. Robert et al. [24] showed that piglets performed more aggressive behavior when they were fostered on day 7 post-partum. Environmental factors could affect the blood stress hormones of pigs [25,26], but few studies on cross-fostering have focused on the blood stress hormones and immunoglobulins of piglets. At present, most research on cross-fostering time reported that late cross-fostering had an adverse effect on the growth performance, mortality and behavior of piglets, but the effect of cross-fostering piglets of different ages still remains to be investigated.

The purpose of this study was (1) to investigate the effect of cross-fostering piglets of different birth weights into new litters on the growth performance of piglets; (2) to determine the effect of cross-fostering piglets of different ages on the growth performance, stress and immunity of these piglets.

## 2. Materials and Methods

The experimental protocols used in this experiment were approved by the Institutional Animal Care and Use Committee of China Agricultural University (Beijing, China) (No. AW09089102-6).

### 2.1. Animals and Experimental Design

This study was conducted on a breeding farm in Guangdong, China, with a herd size of 400 sows, from January to April 2019, and two experiments were conducted. Experimental sows and piglets were purebred Luchuan pigs—this is one of the famous native breeds in China. Luchuan pigs are mainly distributed in southeast China, and they have the characteristics of small size, high productivity, early sexual maturity and good maternity [27]. We analyzed the birth weight of 13,222 piglets from December 2017 to December 2018 and found that the average birth weight of piglets was 0.68 (±0.18) kg. Sows were moved into the farrowing house from day 4 before predicted parturition date and were housed in farrowing crates, which occupied an area of 1.8 m × 0.6 m, and farrowing pens occupied an area of 2.3 m × 1.8 m. The temperature of the farrowing house was maintained at 24–27 °C during the first week after parturition, and 22–24 °C during other experimental periods. All sows were fed two times a day, at 07:30 and 16:30, and received 0.5 kg on day 2 after parturition and then 0.5–1 kg extra per day onwards to a maximum of 5 kg on day 10 after parturition. On day 5 after parturition, male piglets were castrated under analgesia and isoflurane anesthesia. On day 7 after parturition, piglets received piglet feed. Both sows and piglets had free access to water.

In the first experiment, piglets were divided into three different levels, including high birth weight (HBW—>0.8–1.15 kg), intermediate birth weight (IBW—0.6–0.8 kg) and low birth weight (LBW—<0.6–0.35 kg) piglets according to their birth weight. Six piglets (2 HBW piglets, 2 IBW piglets and 2 LBW piglets) were selected from each litter as experimental piglets, and three (1 HBW piglet, 1 IBW piglet and 1 LBW piglet) of the six piglets were fostered piglets and cross-fostered (C-F) at 18–24 h after birth. The rest were not cross-fostered (NC-F) piglets, except for three fostered piglets in the same litter, and they were born on the same day. There were 20 litters (i.e., 20 sows) in the experiment. A total of 120 piglets were classified to one of 6 treatments in a 2 × 3 factorial arrangement. The treatments consisted of piglets without or with cross-fostering (C-F, NC-F) and different birth weights (LBW, IBW, HBW). Each treatment included 20 piglets. The sex of piglets was evenly distributed across fostered and non-fostered experimental piglets (i.e., 8 male HBW fostered piglets and 8 male HBW non-fostered piglets, 7 male IBW fostered piglets and 7 male IBW non-fostered piglets, 4 male LBW fostered piglets and 4 male LBW non-fostered piglets), with 31.7% being males and 69.3% being females. During the experiment, we did not find that sows harmed the piglets and all experimental piglets survived. Twenty sows of parity 3–5 had similar body condition. Each litter involved 12–14 piglets. Piglets were weaned at 23–27 days after farrowing. The average litter size was 11.85 ± 0.24 (mean ± SEM, SEM means standard error of mean) piglets at weaning, and the mortality of piglets was 6.32% during the experiment.

In the second experiment, six sows (parity 3–5) with similar body condition and farrowed on the same day were selected. Each litter included 12–14 piglets. Three female piglets with a birth weight of 0.6–0.85 kg (mean ± SEM, 0.74 ± 0.08 kg) were selected from each litter as experimental piglets. The 18 piglets were grouped to the three treatments: (1) not cross-fostered (NC-F1, n = 6), (2) cross-fostered at 36–48 h after birth (C-F1, n = 6), (3) cross-fostered at 7 day after birth (C-F2, n = 6). A cross-fostered piglet was cross-fostered to another of the experimental litters in exchange for a cross-fostered piglet from this litter. The piglets were weaned at 21 day of age. The average litter size was 12.33 ± 0.42 (mean ± SEM) piglets at weaning, and the mortality of piglets was 7.5% during the experiment.

### 2.2. Measurements

In the first experiment, experimental piglets were weighted individually at birth and weaning. Average daily gain (ADG) was calculated from birth and weaning.

In the second experiment, experimental piglets were weighted individually at 0, 7, 14 and 21 days of age. ADG was calculated for each time period. Suckling positions of experimental piglets (i.e., 1 represented the first teat pair, 2 represented the second teat pair) were recorded two times at 8:30–9:00 and 15:30–16:00 on days 3, 5, 8, 12, 16 and 20 after parturition when suckling behavior occurred. Blood samples were taken from the experimental piglets’ jugular vein at 7:00–9:00 on day 21 after parturition. Blood was collected in a Heparin tube and centrifuged at 3000 r/min for 10 min. Plasma was recovered for the growth hormone (GH), cortisol (COR), alpha-amylase (α-AMY), immunoglobulin A (IgA), immunoglobulin G (IgG), and immunoglobulin M (IgM) assay. GH levels were measured by enzyme immunoassay (Growth hormone EIA kit, Nanjing Jiangcheng Bioengineering Institute, Nanjing, China), and COR levels were measured by enzyme immunoassay (Cortisol EIA kit, DRG International, Springfield, NJ, USA). A-AMY levels were measured by the iodine-starch colorimetric method (Amylase EIA kit, Nanjing Jiangcheng Bioengineering Institute, Nanjing, China). IgA, IgG and IgM levels were measured by turbidimetric inhibition immuno assay (Automatic biochemical analyzer, HITACHI, Tokyo, Japan).

### 2.3. Statistical Analyses

Data analysis was carried out using the statistical software JMP 14.1 (SAS Institute Inc., Cary, NC, USA). All data were tested for normality by the Shapiro–Wilk test. Significant differences were considered at the 95% confidence level (*p <* 0.05). In the first experiment, birth weight (BW), weaning weight (WW) and ADG were analyzed using the linear mixed model, and the sow number is included as a random factor. In the second experiment, body weight (BW1), ADG and suckling position were analyzed as repeated measures by the linear mixed model, with the fixed effect of cross-fostering time, day, and interaction between these two factors [5]. One-way analysis of variance (ANOVA) was used to test the cross-fostering time effects on ADG from 0 day to 21 day, COR, α-AMY, IgA, IgG and IgM of piglets. Correlations between COR, α-AMY, IgA, IgG, IgM and growth performance were analyzed using the Pearson correlation coefficient.

## 3. Results

### 3.1. Birth Weight, Weaning Weight and Average Daily Gain

Significant birth weight class (BWC) effects were exhibited in BW, WW and ADG (*p <* 0.05), and there were no significant effects of treatment and interactions between treatment and BWC in BW, WW and ADG. For LBW piglets and IBW piglets, the WW and ADG were not significantly different between the NC-F group and the C-F group. The ADG of NC-F was significantly higher compared with C-F in the HBW piglets (*p* < 0.05) (Table 1).

### 3.2. Growth Performance 

There were significant effects of treatment and interactions between treatment and day on the body weight (BW1) and ADG (*p* < 0.05). On days 14 and 21, the BW1 of not cross-fostered (NC-F1) and cross-fostered at 36–48 h after birth (C-F1) piglets was higher than cross-fostered at 7 day after birth (C-F2) piglets (*p* < 0.05). The NC-F1 and C-F1 piglets had higher ADG between 7 day of age and 14 day of age and between birth and weaning compared with the C-F2 piglets (*p* = 0.01) (Table 2).

### 3.3. Suckling Positions

A significant treatment (*p* = 0.04) effect was exhibited and significant interactions between treatment and day (*p* = 0.01) were observed in suckling position. On days 3 and 5, there was no difference in the suckling position. The suckling positions of NC-F1 and C-F1 piglets on days 8, 12, 16 and 20 were more forward than the C-F2 piglets (*p* < 0.05) (Table 3).

### 3.4. Plasma Serum Parameters 

Plasma cortisol concentrations of NC-F1 and C-F1 piglets were less than C-F2 piglets (*p* < 0.05) (Table 4). There were no significant effects of different treatments on plasma GH, α-AMY, IgA, IgG and IgM concentrations (*p* > 0.05) (Table 4).

### 3.5. Correlations between Growth Performance and Plasma Serum Parameters

A significant positive correlation was observed between BW1 at day 21 and plasma GH concentration (r = 0.527, *p* < 0.05, r means correlation coefficient), as well as between ADG from day 0 to day 21 and plasma GH concentration (r = 0.488, *p* < 0.05); a significant negative correlation was observed between BW1 at day 21 and plasma COR concentration (r = −0.734, *p* < 0.05), as well as between ADG from day 0 to day 21 and plasma COR concentration (r = −0.736, *p* < 0.05). There was no significant correlation between BW1 at day 21 and plasma α-AMY, IgA, IgG, and IgM (*p* > 0.05), as well as between ADG from day 0 to day 21 and plasma α-AMY, IgA, IgG, and IgM (*p* > 0.05) (Table 5).

## 4. Discussion

A higher concentration of immunoglobulins in sow colostrum within the 12 h period post farrowing was found than at other times [28], and they were better absorbed through the intestinal barrier of piglets during this time [29,30]. In order to ensure that piglets sucked enough colostrum, piglets were cross-fostered more than 12 h after birth in our experiments. Alexopoulos et al. [1] also advised that piglets should stay with their birth sow for at least 12 h. Cross-fostering within 24 h of birth had no effect on the BW1 of non-fostered piglets and fostered piglets [4,5,31]—there was a lack of difference in the WW and ADG of LBW and IBW piglets between treatments in the current study. However, we found that cross-fostering had an adverse effect on the ADG of HBW fostered piglets, which could be due to the fact that HBW fostered piglets fought more and sucked less milk than HBW non-fostered piglets [30]. Piglet size was an important factor for growth and survival [1]. Souza et al. [15] found that LBW fostered piglets missed more nursing episodes when they were mixed with high BW fostered piglets, and HBW piglets vigorously stimulate the udder and suck more milk [32,33]; this may lead to a lower growth performance of LBW piglets than HBW piglets [14,34]. In the present study, we also found that the WW and ADG of LBW piglets (including C-F and NC-F piglets) were lower than HBW piglets.

The age of cross-fostering was an important factor for the growth performance and behavior of fostered piglets. Many authors advised that cross-fostering should be implemented soon after farrowing [35,36,37]. However, some piglets with low growth performance appear in litters during lactation (i.e., over 3 day after parturition), and they may also need to be fostered. Kooij et al. [38] and Wattanaphansak et al. [39] found that cross-fostering on days 2–3 after farrowing had no significant effect on growth performance, whereas Robert et al. [24] and Calderón et al. [8] found that cross-fostering in the first week after farrowing impaired the growth performance of piglets. Our results showed that piglets which were cross-fostered on day 7 after birth had lower BW1 and ADG compared with NC-F1 and C-F1 piglets, which was in line with the above research. In addition, we found that the ADG of C-F2 piglets in the second week after farrowing was lower than other experimental periods. Some studies have reported that the teat order of piglets was developed in the first few days after birth and was relatively stable after one week of birth [40,41], and the stability of teat order had a positive effect on the growth performance of piglets [42]. On days 3 and 5 post-partum, there was lack of difference in the suckling position of piglets among treatments, and when piglets were cross-fostered on day 7 post-partum, the suckling position of C-F2 piglets was more backward compared with the other two treatments in the present study. Huting et al. [14] reported that the BW1 of piglets suckling the anterior teats was higher than piglets suckling the posterior teats. The results demonstrated that cross-fostering on day 7 after farrowing damaged the stability of teat order, and this may lead to the lower growth performance of C-F2 piglets.

In terms of the stress of piglets, plasma COR and α-AMY were selected to evaluate the stress level of piglets [43,44]. Differences were observed such that the COR concentrations of C-F2 piglets were higher than NC-F1 and C-F1 piglets, which indicated that cross-fostering on day 7 after farrowing increased the stress level of piglets. Similar results have been found that the stress level of piglets was increased when unfamiliar piglets were mixed together [45]. Horrell et al. [46] reported that mother-offspring bonds formed early in lactation (i.e., within 3 days after parturition). In fostered piglets removed from their mother to a new environment on day 7 after farrowing, the maternal bond was destroyed, and fostered piglets fought with non-littermates [9,47]. Hence, it is possible that cross-fostering on day 7 after farrowing increased the stress level of fostered piglets. In addition, we found a significant negative correlation between plasma COR concentration and growth performance, which indicated that stress had an adverse effect on the growth performance of piglets. There was no difference in the immunoglobulins level of piglets among treatments.

## 5. Conclusions

Cross-fostering within 24 h of birth has adverse influences on the average daily gain of high birth weight piglets, while it has no negative effect on the average daily gain of low birth weight and intermediate birth weight piglets. Moreover, for intermediate birth weight piglets, late cross-fostering (i.e., on day 7 after farrowing) has negative impacts on the growth performance and teat order of piglets, and it increases the stress level of piglets. We suggest that low birth weight and intermediate birth weight piglets might be more suitable to cross foster as early as possible (i.e., 12–48 h after farrowing) when cross-fostering strategy is implemented.

## Figures and Tables

**Table 1:** The effect of different piglets’ birth weight through cross-fostering on the BW, WW and ADG of piglets.

Item	Treatment	Birth Weight Class (BWC)			SEM	*p*-Values		
		LBW	IBW	HBW		Treatment	BWC	Treatment × BWC
BW (kg)	NC-F	0.46	0.71	0.94	0.02	0.88	<0.01	0.93
	C-F	0.47	0.70	0.94				
WW (kg)	NC-F	3.78	4.46	5.06	0.06	0.19	<0.01	0.53
	C-F	3.68	4.31	4.80				
ADG (g)	NC-F	128.69	145.52	159.70 ^a^	1.72	0.07	<0.01	0.48
	C-F	124.76	139.59	149.58 ^b^				

BW—birth weight; WW—weaning weight; ADG—average daily gain; NC-F—not cross-fostered; C-F—cross-fostered; LBW—low birth weight; IBW—intermediate birth weight; HBW—high birth weight; N _(NC-F, LBW)_ = 20, N _(C-F, LBW)_ = 20, N _(NC-F, IBW)_ = 20, N _(C-F, IBW)_ = 20, N _(NC-F, HBW)_ = 20, N _(C-F, HBW)_ = 20, N means the number of piglets per group; SEM—standard error of mean; ^a, b^—Values with different superscripts differ significantly (*p* < 0.05) among different treatments (columns).

**Table 2:** Effect of different cross-fostering times on the growth performance of piglets.

Item	Day	Treatment			SEM	*p*-Values		
		NC-F1	C-F1	C-F2		Treatment	Day	Treatment × Day
BW1 (kg)	0	0.73	0.77	0.73	0.12	0.04	<0.01	0.02
	7	1.51	1.47	1.47				
	14	2.69 ^a^	2.61 ^a^	1.99 ^b^				
	21	3.58 ^a^	3.50 ^a^	2.73 ^b^				
ADG (g)	0–7	111.07	99.52	104.76	5.82	0.01	0.06	0.03
	7–14	169.52 ^a^	163.33 ^b^	75.17 ^c^				
	14–21	126.43	127.02	105.07				
ADG (g)	0–21	135.67 ^a^	129.96 ^a^	95.00 ^b^	6.46	0.01	-	-

BW1—body weight; ADG—average daily gain; NC-F1—not cross-fostered, N _(NC-F1)_ =6, N means the number of piglets per group; C-F1—cross-fostered at 36–48 h after birth, N _(C-F1)_ = 6; C-F2—cross-fostered at 7 day after birth, N _(C-F2)_ = 6; SEM—standard error of mean; ^a, b, c^—Values with different superscripts differ significantly (*p* < 0.05) among different treatments (rows).

**Table 3:** Effect of different cross-fostering times on the suckling position of piglets.

Item	Day	Treatment			SEM	*p*-Values		
		NC-F1	C-F1	C-F2		Treatment	Day	Treatment × Day
Suckling position	3	3.00	3.08	3.08	0.11	0.04	0.20	0.01
	5	3.33	3.33	3.25				
	8	3.16 ^a^	3.25 ^a^	4.33 ^b^				
	12	2.75 ^a^	3.08 ^a^	4.92 ^b^				
	16	2.92 ^a^	3.12 ^a^	4.92 ^b^				
	20	3.08 ^a^	3.08 ^a^	5.00 ^b^				

NC-F1—not cross-fostered, N _(NC-F1)_ = 6, N means the number of piglets per group; C-F1—cross-fostered at 36–48 h after birth, N _(C-F1)_ = 6; C-F2—cross-fostered at 7 day after birth, N _(C-F__2)_ = 6; SEM—standard error of mean; ^a, b^—Values with different superscripts differ significantly (*p* < 0.05) among different treatments (rows) within the same day.

**Table 4:** Effect of different cross-fostering times on the plasma parameters of piglets.

Plasma Parameters	NC-F1	C-F1	C-F2	SEM	*p*-Value
GH (ng/mL)	1.99	1.90	1.87	0.13	0.93
COR (ng/mL)	14.08 ^a^	15.52 ^a^	17.44 ^b^	0.56	0.03
α-AMY (U/dL)	153.12	148.71	150.85	5.90	0.96
IgA (g/L)	1.04	1.01	0.92	0.03	0.36
IgG (g/L)	9.31	9.52	9.46	0.16	0.87
IgM (g/L)	0.86	0.83	0.77	0.03	0.50

GH—growth hormone; COR—cortisol; α-AMY—alpha-amylase; IgA—immunoglobulin A; IgG—immunoglobulin G; IgM—immunoglobulin M; NC-F1—not cross-fostered, N _(NC-F1)_ = 6, N means the number of piglets per group; C-F1—cross-fostered at 36–48 h after birth, N _(C-F1)_ = 6; C-F2—cross-fostered at 7 day after birth, N _(C-F__2)_ = 6; SEM—standard error of mean; ^a, b^—Values with different superscripts differ significantly (*p* < 0.05) among different treatments (rows).

**Table 5:** Correlation analysis between growth performance and plasma parameters.

Growth Performance	GH	COR	α-AMY	IgA	IgG	IgM
BW1 at day 21	0.527 *	−0.734 *	−0.197	0.132	0.108	0.216
ADG from day 0 to day 21	0.488 *	−0.736 *	−0.211	0.134	0.024	0.133

BW1—body weight; ADG—average daily gain; GH—growth hormone; COR—cortisol; α-AMY—alpha-amylase; IgA—immunoglobulin A; IgG—immunoglobulin G; IgM—immunoglobulin M; NC-F1—not cross-fostered, N = 6; C-F1—cross-fostered at 36–48 h after birth, N = 6; C-F2—cross-fostered at 7 day after birth, N = 6; * *p* < 0.05, (−)denotes a negative association.
